# The Phenomenon of Piebaldism in Sharks: A Review of Global Sightings and Patterns

**DOI:** 10.1002/ece3.71680

**Published:** 2025-06-27

**Authors:** Darren A. Whitehead, Andrea Parmegiani, Jacopo Gobbato, Mohamed Mizyan, Arzucan Askin, Sara Scroglieri, Paolo Galli, Davide Seveso, Simone Montano, Joel H. Gayford

**Affiliations:** ^1^ Shark Research Mexico A.C La Paz Mexico; ^2^ Department of Earth and Environmental Sciences (DISAT) University of Milano—Bicocca Milan Italy; ^3^ MaRHE Center (Marine Research and High Education Center) Magoodhoo Maldives; ^4^ Miyaru – Shark Programme Fuvahmulah Gnaviyani Atoll Maldives; ^5^ University of Dubai Dubai City United Arab Emirates; ^6^ Department of Marine Biology and Aquaculture James Cook University Townsville Queensland Australia; ^7^ Shark Measurements London UK

**Keywords:** Albinism, Elasmobranchii, Evolution, Hypomelanosis, Leucism, Pigmentation

## Abstract

Chromatic disorders in elasmobranchs (sharks and rays) have been reported in several species, but little is known about their true abundance or consequences for survival and fitness. Piebaldism, a form of leucism characterized by partial loss of pigmentation, is among the rarest and least understood chromatic disorders reported in elasmobranchs. We conducted an extensive assessment of published and unpublished cases of piebaldism observed in sharks in the wild. Here, we report the observation of 25 incidences of this condition witnessed in 17 species of sharks from 11 families (Carcharhinidae, Dalatiidae, Galeocerdonidae, Ginglymostomatidae, Hexanchidae, Heterodontidae, Lamnidae, Odontaspidae, Scyliorhinidae, Squatinidae, and Sphyrnidae). The anatomical distribution and severity varied across shark families, with Ginglymostomatidae showing widespread aberrations on the flanks and pectoral fins, while piebaldism in Galeocerdonidae is more restricted. A single Sphyrnidae individual exhibited piebaldism across its body, while in the speciose Carcharhinidae family, piebaldism varied widely in intensity and distribution. We further discuss the taxonomic and geographical distribution of piebaldism reports, the potential fitness consequences, and uncertainty regarding the terminology used to discuss chromatic disorders in elasmobranchs. Finally, we comment on the importance of ecotourism and citizen science for improving our understanding of rare phenotypic conditions in marine megafauna such as elasmobranchs.

## Graphical abstract

Chromatic disorders like piebaldism—a rare form of leucism causing partial pigment loss—have been documented in 25 wild shark cases across 17 species and 11 families, with varying anatomical distribution. This study compiles published and unpublished records to better understand the taxonomic and geographical spread of piebaldism and its potential impact on fitness. It also highlights the key role of ecotourism and citizen science in detecting rare phenotypic traits in elasmobranchs.

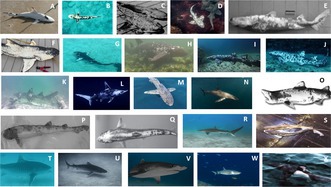

## 1 Introduction

Coloration is an important component of phenotype in most animals, facilitating the transfer of information between conspecifics, competitors, and potential predators (Johnstone 1997). Research into the genetic basis of coloration, its ecological consequences, and evolution across generations has progressed rapidly since the 19th century (Baker and Parker 1979; Cuthill et al. 2017). The spectrum of colors observed in the animal kingdom is now known to be produced by combinations of pigments and nanostructures (Shawkey and D'Alba 2017). Genetically entrained intraspecific variation in coloration is relatively common across the animal kingdom and is frequently adaptive (McKinnon and Pierotti 2010; Roulin 2004). However, an array of chromatic disorders has also been described in animals, where individuals display abnormal pigmentation that appears to serve no adaptive benefit (Lucati and López‐Baucells 2016). Documenting chromatic disorders across animal diversity is key if we are to understand the true genetic basis of coloration and the consequences of disruption to “wild” coloration for ecology and evolution.

Elasmobranchs, comprising sharks, rays, and skates, are a diverse and ancient group of cartilaginous fishes within the class Chondrichthyes. Originating around 383 million years ago (Frey et al. 2019), they have evolved a wide range of adaptations that enable them to thrive in various marine and freshwater environments (Compagno 1999). Elasmobranchs play crucial ecological roles in aquatic ecosystems, namely as apex and mesopredators (Braasch et al. 2009; Stevens et al. 2000; Ebert et al. 2021; Dedman et al. 2024). Their evolutionary success and ecological significance make them a key focus of marine biological research and key indicators of a healthy marine ecosystem. Various pigmentation anomalies have been documented in sharks, apparently resulting from genetically inherited chromatic disorders (Quigley et al. 2018; Ratao et al. 2023; Shipley et al. 2022). In vertebrates, these disorders are typically classified as hypermelanosis or hypomelanosis, an excess or deficiency of pigmentation, respectively (Arronte et al. 2022). The term “melanosis” refers to levels of the pigment melanin, which is among the primary determinants of animal color (Bian et al. 2021).

Hypomelanosis in vertebrates can be categorized broadly as albinism, leucism, and a variation of leucism is defined as piebaldism. Albinism is a genetically inherited hypomelanistic condition characterized by the complete absence of pigmentation in the integumentary system and retina, resulting in an individual lacking dark pigments throughout the entire body, including the eyes (Clark 2002; Ratao et al. 2023). In contrast, leucism is a hypomelanistic disorder where pigmentation is reduced or absent, but the extremities and eyes retain some color representative of the species (Bechtel 1995; Clark 2002; Ramos‐Luna et al. 2022). Piebaldism is a rare autosomal dominant variation of leucism that leads to a partial loss of pigmentation across the body or fins while leaving the eyes normally pigmented. It typically manifests as white or pale patches on the otherwise normally pigmented body of the shark, caused by a genetic mutation affecting the anatomical distribution of melanin (Kelsh et al. 1996; Fertl and Rosel 2009; Leroux et al. 2022; Shipley et al. 2022).

In the wild, piebaldism in sharks is rarely documented and largely unstudied relative to more commonly observed animals, such as terrestrial birds and mammals (Baker 1981; Baker and Lott 1983; Bennett and Cuthill 1994; Crawford 1990; Jensen and Møller 2015). The absence of pigmentation in other animals is frequently linked to various health issues, including deficiencies, malformations, behavioral changes, and reduced survival rates (Corn 1986; Kehas et al. 2005; Perrault et al. 2022; Krecsák 2008; Ratao et al. 2023; Slavík et al. 2015, 2016). However, shark skin depigmentation is poorly studied compared to other aspects of their biology, leading to an incomplete understanding of the causes and consequences of abnormal pigmentation in chondrichthyans. This study aims to consolidate and review all observed instances of piebaldism in sharks, providing a foundational knowledge base for future research and offering insights into this pigmentation anomaly and its potential fitness consequences in these iconic predators.

## 2 Methods

### 2.1 Data Search and Filtering

This study compiled both published and unpublished records of piebaldism observed in sharks from public and scientific observations. Piebaldism is defined as a localized absence of pigmentation, resulting in a variable patchy distribution of dark and white areas (Abreu et al. 2013). Piebaldism is sometimes referred to as partial albinism (consequently some of the reports included here were originally categorized as albinism rather than piebaldism), however, it differs from this condition in that coloration of the eyes is not typically disrupted (Abreu et al. 2013).

A comprehensive evaluation of published studies in peer‐reviewed journals was carried out by exploring scientific literature in web databases such as SCOPUS, Google Scholar, and Web of Science. During our search for relevant papers, we utilized various combinations of keywords, including: “piebaldism”, “sharks”, “cartilaginous fish”, “elasmobranch”, “albinism”, “leucism”, “chromatic disorders”, “pigmentation anomalies” along with phrases like “rare sightings” and “first reports”. Following this, data regarding the species, sex, location, year of record, and the type of record (e.g., dive observation, public observation, fishing interaction) were extracted.

Upon finding a scientific paper, we explored its references and citation records to uncover additional relevant sources. Unpublished records were gathered from a variety of platforms, including websites, Citizen Science platforms, social media, and personal communications. We extracted the same sighting metadata (species, sex, location, etc.) from these unpublished sources as we did from the published records. Unverifiable records, or those where chromatic disorders other than true piebaldism (e.g., leucism, or partial/complete albinism) could not be ruled out, were excluded.

## 3 Results

### 3.1 Piebaldism Sightings in Sharks

A total of 25 reports of piebaldism in sharks were identified spanning 17 species across 7 different orders—Carcharhiniformes, Heterodontiformes, Hexanchiformes, Lamniformes, Orectolobiformes, Squatiniformes, and Squaliformes—and covering 11 families: Carcharhinidae, Dalatiidae, Galeocerdidae, Ginglymostomatidae, Heterodontidae, Hexanchidae, Lamnidae, Odontaspididae, Scyliorhinidae, Squatinidae, and Sphyrnidae (Table 1). Comparing the prevalence of piebaldism across these clades, the order Carcharhiniformes showed the highest number of recorded cases, with 10 species affected. Within this order, the family Carcharhinidae (requiem sharks) was the most impacted, accounting for 7 species. In contrast, each of the other nine families had only a single species affected (Table 1). It is important to note that this pattern may reflect sampling bias rather than a true biological prevalence, as Carcharhinidae includes many coastal and commercially important species that are more frequently encountered in fisheries bycatch and scientific surveys. Collectively, records date back to 1952, with the most recent observations occurring in September 2024. The geographic distribution of these sightings was broad but biased, with the majority of observations concentrated in the Indian Ocean. This was followed by a significant number in the Atlantic Ocean and the Mediterranean Sea, while the Eastern Pacific Ocean and other regions had fewer sightings overall. This uneven spatial pattern likely mirrors global disparities in sampling effort, with higher observation frequencies in regions with more active fisheries, tourism, or scientific monitoring programs. Additionally, there were isolated observations recorded in the Gulf of Mexico, the Caribbean Sea, the Irish Sea, and the Arabian Sea.

**TABLE 1:** Complete overview of all sightings.

Sighting	Common name	Species	Sighting type	Location	Year	Source
A	Oceanic black tip shark	*Carcharhinus limbatus* (Müller & Henle, 1839)	Fishing Interaction	Texas, USA	2015	Media Report
B	Blacktip Reef shark	*Carcharhinus melanopterus* (Quoy & Gaimard, 1824)	Observation	Maldives	2023	Social media mining
C	Broudnose Sevengill shark	*Notorynchus cepedianus* (Péron, 1807)	Fishing Interaction	Monterey Bay, California, USA	1952	Published Herald, E.S. (1953)
D	California Horn shark	*Heterodontus francisci* (Girard, 1855)	Dive Sighting	La Jolla, California, USA	2019	Published Skelton et al. (2024)
E	Kitefin shark	*Dalatias licha* (Bonnaterre, 1788)	Fishing Interaction	Genoa, Italy	2003	Published Bottaro et al. (2008)
F	Lemon shark	*Negaprion brevirostris* (Poey, 1868)	Fishing Interaction	Florida, USA	2023	Social media/Media Report
G	Nurse shark	*Ginglymostoma cirratum* (Bonnaterre, 1788)	Dive Sighting	Black Point, Bahamas	2013	Social media
H	Nurse shark	*Ginglymostoma cirratum* (Bonnaterre, 1788)	Dive Sighting	Maio Island, Cabo Verde	2015	Published Ratao et al. (2023)
I	Nurse shark	*Ginglymostoma cirratum* (Bonnaterre, 1788)	Dive Sighting	Turks & Caicos islands	2016	Media Report
J	Nurse shark	*Ginglymostoma cirratum* (Bonnaterre, 1788)	Dive Sighting	Utila, Honduras	2022	Published Shipley et al. (2022)
K	Nurse shark	*Ginglymostoma cirratum* (Bonnaterre, 1788)	Dive Sighting	East Bahia Honda, Florida Keys, USA	2023	Published Becker et al. (2023)
L	Scalloped Hammerhead shark	*Sphyrna lewini* (Griffith & Smith, 1834)	Dive Sighting	Fotteyo, Maldives	2014	Unpublished
M	Silky shark	*Carcharhinus falciformis* (Müller & Henle, 1839)	Observation	San Jose Del Cabo, Mexico	2024	Unpublished
N	Silky shark	*Carcharhinus falciformis* (Müller & Henle, 1839)	Observation	Cabo San Lucas, Mexico	2024	Unpublished
O	Smalltooth Sandtiger shark	*Odontaspis ferox* (Risso, 1810)	Fishing Interaction	Keeling Islands, Greece	2004	Published Fergusson et al. (2008)
P	Small Spotted Catfish shark	*Scyliorhinus canicula* (Linnaeus, 1758)	Fishing Interaction	Tunisia	2009	Published Mnasri et al. (2010)
Q	Spadenose shark	*Scoliodon laticaudus* (Müller & Henle, 1839)	Fishing Interaction	Mangalore, India	2006	Published Veena et al. (2011)
R	Spinner shark	*Carcharhinus brevipinna* (Müller & Henle, 1839)	Dive Sighting	Hulhumale, Maldives	2024	Unpublished
S	Spotted Dogfish shark	*Scyliorhinus canicula* (Linnaeus, 1758)	Fishing Interaction	Irish Sea	2017	Published Quigley et al. (2018)
T	Tiger shark	*Galeocerdo cuvier* (Péron & Lesueur, 1822)	Dive Sighting	Hulhumale, Maldives	2023	Unpublished
U	Tiger shark	*Galeocerdo cuvier* (Péron & Lesueur, 1822)	Dive Sighting	Hulhumale, Maldives	2024	Unpublished
V	Tiger shark	*Galeocerdo cuvier* (Péron & Lesueur, 1822)	Dive Sighting	Hulhumale, Maldives	2024	Unpublished
W	Whitetip Reef shark	*Triaenodon obesus* (Rüppel, 1837)	Dive Sighting	Miyaru Kandu, Maldives	2019	Unpublished
X	Great White shark	*Carcharodon carcharias* (Linnaeus, 1758)	Observation	Saros Bay Turkey	2020	Published Kabasakal (2020)
Y	Angel Shark	*Squatina Squatina* (Linnaes, 1758)	Report	Gran Canaria	2021	Published Jimenez‐Alvarado et al. (2023)

### 3.2 Distribution and Severity of Piebaldism

**FIGURE 1:**
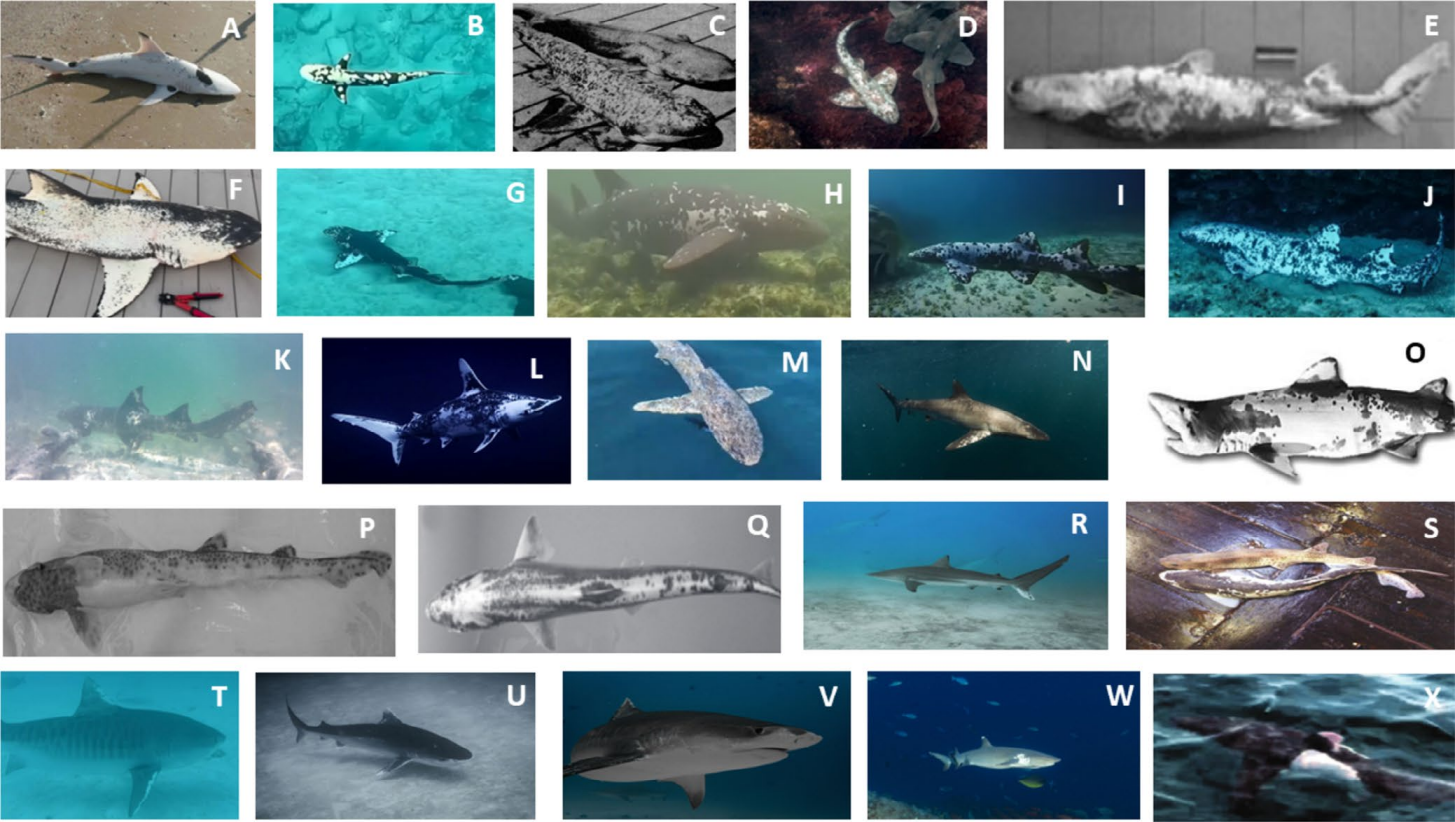
Patterns of Piebaldism across records of shark species.

The anatomical distribution and severity of piebaldism varied among species and families (Figure 1). In the Ginglymostomatidae family, individuals consistently exhibited color aberrations that extended across their entire flanks and both pectoral fins (Figure 1G,K). In contrast, members of the Galeocerdonidae family consistently displayed abnormalities only on the leading edges of their pectoral and first dorsal fins, with no noticeable markings elsewhere (Figure 1T–V). The single individual from the Sphyrnidae family showed clear piebaldism across its entire body and fins, particularly on the sides of its head and caudal fin (Figure 1L), where pigmentation was absent compared to the rest of its flanks similar to individuals observed with Scyliorhinidae (Figure 1S), Dalatidae (Figure 1E), Hexanchidae (Figure 1C) and Odontaspidae (Figure 1O). Among the Carcharhinidae family (Figure 1A,B,M,N,R,X), which has the most diverse range of species, individuals exhibited a wide variety of piebaldism intensities. Some individuals showed similar patterns or tendencies across their pectoral fins and bodies, while others displayed abnormalities only in specific areas, such as the lower part of the caudal fin or around the gills.

## 4 Discussion

Our goal was to collect information on this rare chromatic disorder in sharks and establish a comprehensive global baseline regarding the prevalence of these pigmentation anomalies among this group of marine predators. Cumulatively, existing reports of piebaldism in sharks cover six of the nine extant orders (Weigmann et al. 2011), with only Echinorhiniformes and Pristiophoriformes lacking confirmed reports, which may be due to the fact that they are seldomly caught in fisheries and for the most part inhabit deep‐sea environments or remote benthic zones (Table 1). Indeed, this absence is likely due to limited sampling opportunities: Echinorhiniformes are deep‐water species that are rarely observed or captured, while Pristiophoriformes (sawsharks) are relatively rare and have restricted distributions, making encounters uncommon in most survey and fishery contexts. The majority of existing reports are from Carcharhiniformes, which is not overly surprising given that this is the most speciose order of sharks (Ebert et al. 2021). Moreover, considering their spatial distribution and habitat usage, Carcharhiniformes species are frequently observed in coastal and reef environments by recreational fishers and ecotourists, further increasing the likelihood of encountering individuals with rare chromatic disorders, and consequently reported. Chance observations by recreational fishers or ecotourists would be far less likely in the case of taxa that occur exclusively in pelagic or deep‐water environments. Consequently, on the basis of the limited existing data, there is no reason to suggest that piebaldism is more prevalent in some shark lineages than others, and we consider the taxonomic bias observed to be merely symptomatic of biased sampling.

Piebaldism remains a rare and understudied condition in sharks across various orders and species. Genetic studies in zebrafish ( *Danio rerio* ) have established that one of the genes implicated in this disorder is the *kit* (kit receptor tyrosine kinase) gene, mutations of which led to dysfunctions in melanophores, disrupting their development, migration, and survival (Parichy et al. 1999). Infectious diseases caused by parasites or bacteria can also change skin appearance by targeting pigment cells or causing lesions. Nutritional deficiencies, particularly a lack of essential vitamins and minerals, can adversely affect skin health and pigmentation. Furthermore, environmental stressors, such as fluctuations in temperature and salinity, as well as exposure to chemicals and pollution, particularly in urban areas, may be drivers of changes in skin coloration. Understanding the multifactorial nature of skin coloration in sharks is essential for comprehensively addressing the causes of piebaldism and other pigmentation anomalies. Moreover, recent studies have linked skin pigmentation disorders in fishes to exposure to toxic elements such as copper and mercury (Nur et al. 2019; Qu et al. 2023). Considering both the genetic basis and the potential ecotoxicological factors that may trigger this effect, it would be valuable for future research to investigate the genetic basis of piebaldism in elasmobranchs, and the extent to which observed cases may be explained by environmental pollution. At least four of the reported cases (individuals of *G. cuvier* and *C. brevipinna* sighted in Hulhumale, see Figure 1) were observed in close proximity to residential/industrial areas, which may be consistent with elevated levels of environmental pollutants.

Although depigmentation in sharks can occur on the flanks, head, fins, and dorsal surface, no current studies have systematically examined which regions of the body are most susceptible to this condition. Given the limited sample size in this study and the absence of genetic analyses, it is not yet possible to determine whether specific areas of the body are more prone to piebaldism or what factors may be influencing the pattern and extent of this condition. Further research, including genetic analyses on larger sample sizes, is needed to provide clearer insight into the factors underlying variation in the anatomical location and intensity of observed piebaldism.

Besides the frequency and taxonomic distribution of piebaldism in sharks, perhaps the most substantial unanswered question regards the consequences (or lack thereof) of piebaldism for individual fitness. As many shark species rely on camouflage to successfully hunt and avoid predation, it has been speculated that abnormal pigmentation could impact survivability by making individuals more conspicuous to both predators and prey (Bottaro et al. 2008). Beyond survival, it is also plausible that abnormal pigmentation could influence growth rates, fecundity, or other components of individual fitness (Denson and Smith 1997; Dubovskiy et al. 2013). For example, reduced foraging efficiency due to impaired camouflage could limit energy intake and consequently slow growth. In terms of reproduction, conspicuous coloration might alter intraspecific interactions, such as mate recognition or selection, particularly in species that rely on visual cues during courtship (Pratt and Carrier 2005). Though speculative in the context of sharks, pigmentation‐driven sexual selection has been documented in other taxa, and may warrant consideration as a potential, but currently untested, factor influencing fitness in elasmobranchs (Warner et al. 1975; Ball 2024; Dijkstra et al. 2024; Tripathy et al. 2025). However, none of the reported cases of piebaldism in sharks reviewed in this study appear to be associated with any abnormal morphology (Figure 1). In cases where video footage was available, no unusual behavior was observed, and the sharks appeared to swim and interact normally with their environment. While the lack of age data limits precise size assessments, there was no evidence of obvious growth anomalies relative to conspecifics observed in similar contexts. The observation of multiple adult piebald sharks also indicates that piebaldism likely does not drastically reduce survivorship, at least in the few species in which it has been identified. It has previously been suggested that larger‐bodied generalist sharks may not suffer fitness consequences from piebaldism due to a relative lack of predators (Shipley et al. 2022), however, this does not explain observations of piebaldism in smaller species with many natural predators, such as *S. canicula* or *H. francisci* . Ultimately, we are fundamentally limited in our ability to determine the fitness costs of piebaldism in sharks as any individuals that do succumb to predation as a direct result of abnormal pigmentation are consumed and hence impossible to observe. Although estimating an expected baseline prevalence of piebaldism across shark species using comparative data from other taxa could, in principle, provide a framework to assess whether observed cases are under‐ or overrepresented, this would require genetic data and robust assumptions about developmental mutation rates across taxa, both of which are currently unavailable for sharks. Sharks held in laboratories and aquaria provide one potential solution to this issue, but to date, no cases of piebaldism have been published from such facilities.

Moreover, while the precise causes and consequences of piebaldism in sharks remain unclear, it is worth considering whether certain pigmentation anomalies may persist in populations due to neutral or even mildly beneficial effects. For example, in species like the oceanic whitetip shark ( *Carcharhinus longimanus* ), natural markings include irregular light patches on the tips of fins and body, which bear a superficial resemblance to some piebald patterns documented in this study. Although speculative, it is possible that ancestral piebald‐like traits conferred some ecological or social advantage, such as camouflage against the dappled light of the open ocean. In this context, low‐level depigmentation may not always be deleterious and could, under certain environmental or behavioral conditions, be selectively neutral or even advantageous (Myrberg 1990; Wilson and Martin 2001). This idea aligns with the broader concept that pigmentation anomalies are not always negative, and their persistence may reflect a complex interplay between genetic drift, selection, and context‐dependent fitness outcomes. Further comparative and genomic studies could help clarify whether piebaldism in sharks occasionally crosses the threshold from neutral anomaly to adaptive trait.

One notable and recurring issue in the shark pigmentation literature is uncertainty around terminology. Until recently, the term albinism was frequently used to refer to any loss of pigmentation, regardless of its intensity or genetic underpinnings (Clark 2002). This has resulted in many cases of leucism (and piebaldism) being mistakenly referred to as cases of complete or partial albinism (see reclassification of misidentified chromatic disorders in Skelton et al. 2024). Misuse of terminology and misclassification of chromatic disorders is a pervasive issue that has hampered research into pigmentation across different vertebrate lineages (Borteiro et al. 2021). Given that albinism and leucism can have distinct genetic underpinnings in other vertebrates, it is crucial to avoid misnomers when classifying chromatic disorders in sharks. All future studies should study ocular pigmentation to distinguish between true albinism and leucism and study the entire external surface of individuals to distinguish between full and partial albinism and between leucism and piebaldism. Whilst the terms piebaldism and partial leucism are interchangeable, neither is interchangeable with albinism or partial albinism, as these represent genetically and phenotypically distinct conditions.

We encourage scientific societies and the expanding community of divers and ocean users to share their knowledge and data by reporting any sightings of these anomalies they encounter. There is undoubtedly a substantial geographical and taxonomic bias in existing reports of piebaldism and chromatic disorders in general. This bias will only be overcome through collaboration between researchers, commercial, artisanal, and recreational fishers, as well as with the collaboration of ecotourists, divers, and citizen scientists. Indeed, citizen science projects have been key to the study of chromatic disorders and pigmentation in other taxa (Aguillon and Shultz 2023; Drury et al. 2019; Paiva et al. 2023). Whilst few of the reports in this study originated from citizen science projects, collaborations between researchers and the public have previously provided key insight into shark behavior and ecology (Séguigne et al. 2023; Whitehead and Gayford 2023; Parmegiani et al. 2023; Gobbato et al. 2024). This is reflected also by our findings showing an increase in sightings in recent years, with 2024 recording the highest number of sightings. This surge is likely linked to the rise of citizen science and the growing popularity of recreational diving, engaging more people in marine ecotourism and documenting their experiences through social media and other platforms, which in turn contributes to a higher volume of recorded sightings (Gibson et al. 2019; Bargnesi et al. 2020). Indeed, among the recorded species, *G. cirratum* stands out as the most frequently sighted, as one of the most likely interacting species in marine ecotourism. In contrast, earlier sightings, such as those from the 1950s, reflect a more limited scope of shark documentation, often tied to scientific publications or isolated fishing interactions. Today, however, the increased engagement of the public in marine conservation and documentation efforts has broadened the scope of our understanding of shark populations globally, reflecting the growing awareness of marine conservation and the role that both professionals and citizen scientists play in enhancing our understanding of shark ecology and biology. There is thus hope that future increases in the user bases of shark‐focused citizen science projects may help improve our understanding of chromatic disorders. To support this, we encourage divers, underwater photographers, and ocean users to report sightings through established platforms such as iNaturalist, SharkPulse, GBIF.org, or local biodiversity monitoring apps, and to include details on pigmentation anomalies when possible. Clear documentation, including photographs, videos, date, location, and depth, can greatly enhance the value of these records for scientific analysis. Expanding this participatory approach may further close taxonomic and geographic gaps in the detection of piebaldism and other rare phenotypes.

## 5 Conclusions

Our comprehensive assessment of piebaldism in sharks reveals a notable prevalence of this rare chromatic disorder across various species and families, highlighting its potential underrepresentation in the existing literature. While our findings indicate that piebaldism is widespread, particularly within the Carcharhinidae family, the implications of this condition for individual fitness and survival remain largely unexplored. Given the potential ecological consequences, further research is essential to understand how abnormal pigmentation may influence predator–prey interactions and overall fitness. Collaboration with the broader community, including citizen scientists and divers, will be crucial in expanding our knowledge and addressing the geographical and taxonomic biases evident in current reports. By fostering such partnerships, we can enhance our understanding of piebaldism and its role within the dynamic ecosystems inhabited by these remarkable elasmobranchs.

Although many questions remain unanswered, our study highlights the potential of this research area and lays the groundwork for future discoveries. While recent advances in understanding the genetics of coloration have provided a foundation for studying chromatic disorders, much remains to be uncovered about the environmental and genetic factors driving piebaldism in sharks. Therefore, we believe that future analysis should also investigate the environmental and ecotoxicological aspects of this phenomenon, especially for piebald individuals frequently observed at provisioning sites and in close proximity to urban areas. In a globally changing climate, sharks face multiple stressors including increasing polluted environments and temperature shifts affecting their habitats. Further analysis of these rarely sighted individuals may offer valuable insights into their ecology and adaptation to the environments in which they live.

The rise of citizen science and recreational diving has significantly increased the visibility of shark sightings, offering an invaluable opportunity to gather more data on these anomalies. As seen with *Galeocerdo cuvier* and *Ginglymostoma cirratum* , commonly sighted species in ecotourism, engaging the general public in research will be essential to uncover the frequency and potential consequences of piebaldism in these and other species. Moving forward, a multidisciplinary approach combining genetics, ecotoxicology, and citizen science could provide critical insights into the causes and ecological implications of pigmentation disorders in sharks, ultimately enhancing our broader understanding of elasmobranch anomalies.

## Author Contributions

**Darren A. Whitehead:** conceptualization (lead), data curation (equal), formal analysis (equal), investigation (equal), supervision (equal), validation (equal), writing – original draft (equal), writing – review and editing (equal). **Andrea Parmegiani:** formal analysis (equal), investigation (equal), visualization (equal), writing – original draft (equal), writing – review and editing (equal). **Jacopo Gobbato:** data curation (equal), formal analysis (equal), investigation (equal), writing – original draft (equal), writing – review and editing (equal). **Mohamed Mizyan:** formal analysis (equal), investigation (equal), writing – original draft (equal). **Arzucan Askin:** formal analysis (equal), investigation (equal), writing – original draft (equal), writing – review and editing (equal). **Sara Scroglieri:** formal analysis (equal), investigation (equal), writing – original draft (equal), writing – review and editing (equal). **Paolo Galli:** supervision (equal), validation (equal). **Davide Seveso:** supervision (equal), validation (equal), writing – review and editing (equal). **Simone Montano:** supervision (equal), validation (equal), writing – review and editing (equal). **Joel H. Gayford:** data curation (equal), formal analysis (equal), investigation (equal), writing – original draft (equal), writing – review and editing (equal).

## Conflicts of Interest

The authors declare no conflicts of interest.

## Supporting information


Data S1.

## Data Availability

All the required data are uploaded as Supporting Information.
