# Correction to “The Phenomenon of Piebaldism in Sharks: A Review of Global Sightings and Patterns”

**DOI:** 10.1002/ece3.71827

**Published:** 2025-07-17

Whitehead, D.A., Parmegiani, A., Gobbato, J., et al. (2025). The Phenomenon of Piebaldism in Sharks: A Review of Global Sightings and Patterns. *Ecology and Evolution*, 15 no. 7: e71680. https://doi.org/10.1002/ece3.71680.

After publication, we became aware of an attribution error concerning an image included in Table 1. This mistake was entirely unintentional and occurred during the exchange of information and the preparation of the table between co‐authors. Specifically, we need to amend the **third entry of the table** by changing the **“Location”** from “Monterey Bay, California, USA” to **“Hunter's Point, California, USA”** and the **“Resource”** from “Published Herald, E. S. (1953)” to **“Published Ebert, D. (1985)”**.

The complete reference that should be added to the References section is:

**Ebert, D. (1985) Color variation in the Sevengill shark, *Notorynchus maculates Ayres*, along the California coast. *California Fish and Game*, 71(1): 53–59**.

We have already clarified the situation directly with the rightful author, who has kindly understood our position. Nevertheless, we wish to formally correct the publication to ensure proper attribution and uphold the integrity of the article.

We apologize for this error.

