# Supplementary material for: The Phenomenon of Piebaldism in Sharks: A Review of Global Sightings and Patterns
Source: Ecol Evol. 2025 Jun 27;15(7):e71680. doi: 10.1002/ece3.71680 (PMC12204725; doi:10.1002/ece3.71680)

**SUPPLEMENTARY**

RAW DATA

Table.1 complete overview of all sightings

| **Sighting** | **Common Name** | **Species** | **Sighting type** | **Location** | **Year** | **Source** |
| --- | --- | --- | --- | --- | --- | --- |
| A | Oceanic black tip shark | *Carcharhinus limbatus* | Fishing Interaction | Texas, USA | 2015 | Media Report |
| B | Blacktip Reef shark | *Carcharhinus melanopterus* | Observation | Maldives | 2023 | Social media mining |
| C | Broudnose Sevengill shark | *Notorynchus cepedianus* | Fishing Interaction | Monterey Bay, California, USA | 1952 | Published  Herald, E.S. 1953 |
| D | California Horn shark | *Heterodontus francisci* | Dive Sighting | La Jolla, USA | 2019 | Published  Skelton et al., 2024 |
| E | Kitefin shark | *Dalatias licha* | Fishing Interaction | Genoa, Italy | 2003 | Published  Bottaro et al., 2008 |
| F | Lemon shark | *Negaprion brevirostris* | Fishing Interaction | Florida, USA | 2023 | Social media/ Media Report |
| G | Nurse shark | *Ginglymostoma cirratum* | Dive Sighting | Black Point, Bahamas | 2013 | Social media |
| H | Nurse shark | *Ginglymostoma cirratum* | Dive Sighting | Maio Island,  Cabo Verde | 2015 | Published,  Ratão et al., 2023 |
| I | Nurse shark | *Ginglymostoma cirratum* | Dive Sighting | Turks & Caicos islands, | 2016 | Media Report |
| J | Nurse shark | *Ginglymostoma cirratum* | Dive Sighting | Utila, Honduras | 2022 | Published  Shipley et al.,2022 |
| K | Nurse shark | *Ginglymostoma cirratum* | Dive Sighting | East Bahia Honda, Florida Keys, USA | 2023 | Published  Becker etal.,2023 |
| L | Scalloped Hammerhead shark | *Sphyrna lewini* | Dive Sighting | Fotteyo, Maldives | 2014 | Unpublished |
| M | Silky shark | *Carcharhinus falciformis* | Observation | San Jose Del Cabo, Mexico | 2024 | Unpublished |
| N | Silky shark | *Carcharhinus falciformis* | Observation | Cabo San Lucas, Mexico | 2024 | Unpublished |
| O | Smalltooth Sandtiger shark | *Odontaspis ferox* | Fishing Interaction | Keeling Islands, Greece | 2004 | Published  Fergusson et al., 2007 |
| P | Small Spotted Catfish shark | *Scyliorhinus canicula* | Fishing Interaction | Tunisia | 2009 | Published  Mnasri et al., 2010 |
| Q | Spadenose shark | *Scoliodon laticaudus* | Fishing Interaction | Mangalore, India | 2006 | Published  Veena et al., 2011 |
| R | Spinner shark | *Carcharhinus brevipinna* | Dive Sighting | Hulhumale, Maldives | 2024 | Unpublished |
| S | Spotted Dogfish shark | *Scyliorhinus canicula* | Fishing Interaction | Irish Sea | 2017 | Published  Quigley et al., 2018 |
| T | Tiger shark | *Galeocerdo cuvier* | Dive Sighting | Hulhumale, Maldives | 2023 | Unpublished |
| U | Tiger shark | *Galeocerdo cuvier* | Dive Sighting | Hulhumale, Maldives | 2024 | Unpublished |
| V | Tiger shark | *Galeocerdo cuvier* | Dive Sighting | Hulhumale, Maldives | 2024 | Unpublished |
| W | Whitetip Reef shark | *Triaenodon obesus* | Dive Sighting | Miyaru Kandu, Maldives | 2019 | Unpublished |
| X | Great White shark | *Carcharodon carcharias* | Observation | Saros Bay Turkey | 2020 | Published  Kabaskal et al., 2020 |

Figure 1.


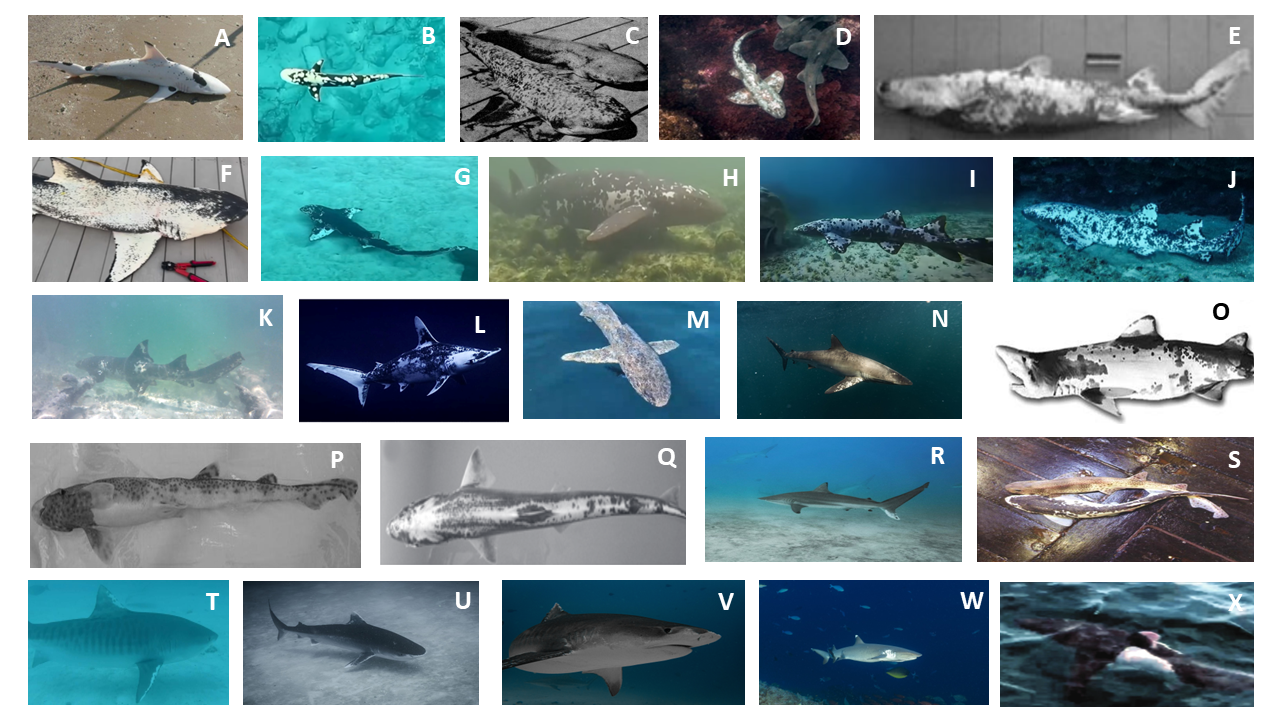

Supplement: Supplementary file 1 — Data S1. [file ECE3-15-e71680-s001.docx]
